# Biological Roles and Delivery Strategies for Ions to Promote Osteogenic Induction

**DOI:** 10.3389/fcell.2020.614545

**Published:** 2021-01-14

**Authors:** Elia Bosch-Rué, Leire Diez-Tercero, Barbara Giordano-Kelhoffer, Luis M. Delgado, Begoña M. Bosch, Mireia Hoyos-Nogués, Miguel Angel Mateos-Timoneda, Phong A. Tran, Francisco Javier Gil, Roman A. Perez

**Affiliations:** ^1^Bioengineering Institute of Technology, Universitat Internacional de Catalunya, Barcelona, Spain; ^2^Centre for Biomedical Technologies, Queensland University of Technology (QUT), Brisbane, QLD, Australia; ^3^Interface Science and Materials Engineering Group, School of Mechanical, Medical and Process Engineering, Queensland University of Technology, Brisbane, QLD, Australia

**Keywords:** bone, scaffolds, tissue engineering, biomaterials, tissue regeneration, therapeutic ions

## Abstract

Bone is the most studied tissue in the field of tissue regeneration. Even though it has intrinsic capability to regenerate upon injury, several pathologies and injuries could hamper the highly orchestrated bone formation and resorption process. Bone tissue engineering seeks to mimic the extracellular matrix of the tissue and the different biochemical pathways that lead to successful regeneration. For many years, the use of extrinsic factors (i.e., growth factors and drugs) to modulate these biological processes have been the preferred choice in the field. Even though it has been successful in some instances, this approach presents several drawbacks, such as safety-concerns, short release profile and half-time life of the compounds. On the other hand, the use of inorganic ions has attracted significant attention due to their therapeutic effects, stability and lower biological risks. Biomaterials play a key role in such strategies where they serve as a substrate for the incorporation and release of the ions. In this review, the methodologies used to incorporate ions in biomaterials is presented, highlighting the osteogenic properties of such ions and the roles of biomaterials in controlling their release.

## Introduction

Bone is a complex and hierarchical organ that is in constant remodeling depending on the specific macro- and microenvironments. The main function of bone tissue is to provide mechanical stability to the body and protect the main organs (Florencio-Silva et al., 2015). Moreover, it has a high turnover which helps maintaining an ion balance in the body. Diseases or traumatic injuries can compromise bone function. For this purpose, it is of great importance to restore the lost functionality in a short and efficient manner. The gold standard has been the use of natural grafts (such as auto- and allografts), although their limited availability and possible transmission of diseases have limited their applications (Younger and Chapman, 1989; Betz, 2002). With this in mind, there is a great need to develop novel synthetic grafts that may provide similar functions to those of natural grafts while avoiding the possible related issues (Moore et al., 2001).

For successful bone regeneration, it is important that the bone substitute mimics to the highest extent the highly orchestrated bone regeneration steps (Perez et al., 2015b). Novel strategies involve the continuous adaptation of the inserted matrices to trigger a series of biological processes that may ultimately lead to bone regeneration (Pérez et al., 2013). These processes will mainly stimulate cells, through specific intrinsic features provided by the biomaterial and/or the release of extrinsic factors allocated within the substrate, such as molecules with biological activity (Perez et al., 2016). Among the different steps, the most critical are mitigation of the possible bacterial infection, the control of the initial inflammatory response, the attraction of cells to the site of defect through the formation of blood vessels, and the final maturation of the bone tissue (Perez et al., 2015b).

The different processes have been generally modulated by a combinatorial approach of intrinsic and extrinsic features of the defined biomaterials. Intrinsic properties, mainly the mechanical properties (both at a local and bulk-level), the surface roughness or the surface charge, are common parameters that can be tuned by a proper design (Navarrete et al., 2017). On the contrary, extrinsic factors are based on the use of biologically active molecules (such as growth factors) and peptides that can exert a positive response on tissue regeneration (Wang Z. et al., 2017). These extrinsic biologically active molecules have shown great potential *in vitro*. However, controlling their adequate delivery as well as their low half-life once implanted in the defect area are major hurdles for their clinical/industrial translation. To this purpose, the use of ions (mainly metal ions) is an attractive option because their therapeutic effect is well-known, they have an increased stability and, in terms of safety, they have lower risk than the use of biomolecules (Mouriño et al., 2012; Perez et al., 2015b). These therapeutic ions released from biomaterials can modulate the tissue regeneration steps in a similar way to biologically active molecules.

Hence, the scope of this review is to describe the vast possibilities of ion incorporation within biomaterials. The review will cover the different possible methodologies of ion incorporation within ceramic, metallic, and polymeric materials and the effect that the different methodologies will have on their final release. The attention will be given to their osteogenic properties, while the antibacterial and angiogenic properties have been reviewed elsewhere (Hoppe et al., 2011; Kaya et al., 2018).

## Bone Regeneration Steps

**Figure 1:**
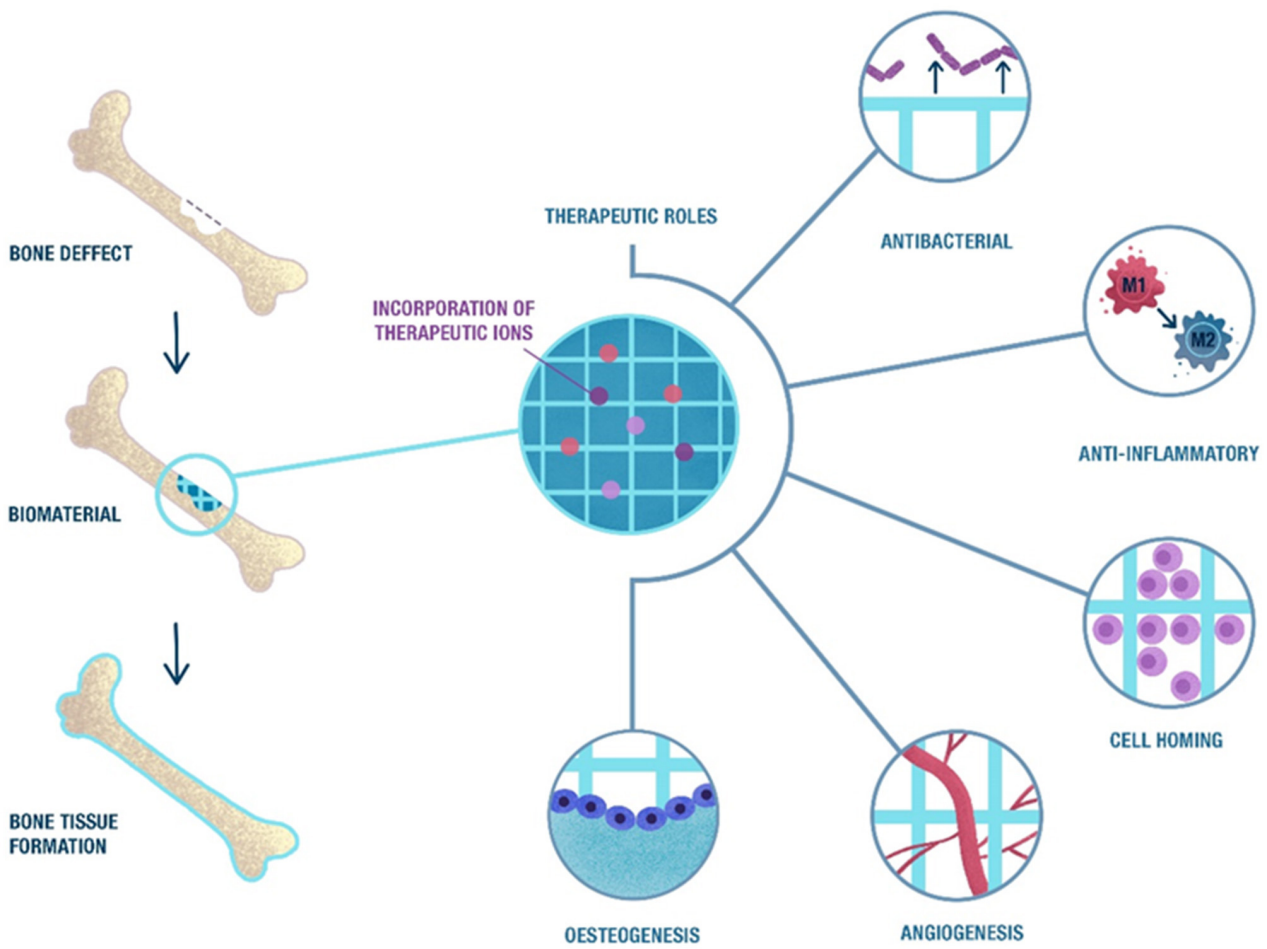
Schematic representation of the biological phases that take place during bone healing in which ions may have a therapeutic role. The natural bone healing starts with a pro-inflammatory response, followed by stem cell homing and the formation of new blood vessels. This process ends with the differentiation of stem cells into osteocytes and the formation of new bone. The release of ions from scaffolds may induce a therapeutic effect in these processes, combined with an anti-bacterial effect that may reduce the risk of infection after implantation.

Once a biomaterial is implanted, a dynamic interaction between the biomaterial surface and the plasma proteins takes place and a temporary matrix around the biomaterial or implanted device is formed; this phenomenon is known as the Vroman effect (Vroman et al., 1980). The initial protein adsorption depends on the surface properties of the biomaterial, which relate to wettability, surface charge, topography or stiffness, among others (Hallab et al., 2001; MacDonald et al., 2002; Xu and Siedlecki, 2007; Shiu et al., 2014), which modulate the initial cell response and subsequent bone healing phases. Bone healing is a complex biological process that is controlled by several growth factors, cytokines, cell types, and mechanical stimuli that direct the different overlapping phases, mainly haemostasis, acute inflammation, progenitor and stem cell homing, angiogenesis, osteogenic differentiation, and mineralisation (Perez et al., 2015b). The last step can be divided in mainly an osteogenic differentiation of stem cells followed by the mineralisation of the differentiated cells as described below. The role of ions has been proved in many of the bone regeneration processes, primarily acting as enzyme co-factors and, therefore, they influence the different signaling pathways (Zhi Lin Sun and Hanks, 1997). The potential role of ions is summarized in Figure 1. Understanding the different phases that take place during bone regeneration might be useful for the design of biomaterials that can deliver cues at specific time points.

### Osteogenic Differentiation

Following stem cell homing, mesenchymal stromal cells (MSCs) proliferate and sequentially differentiate into chondroblasts and osteoblasts. Osteogenic differentiation only happens under specific microenvironments and stimuli, because MSCs have the ability to differentiate into other tissues, such as cartilage and fat (Dominici et al., 2006). Several different factors are implicated in this osteogenic differentiation. For example, physical cues such as stiffness or topographies have been shown to modulate MSCs differentiation (Engler et al., 2006; Dobbenga et al., 2016). On the other hand, growth factors such as fibroblast growth factors (FGFs), bone morphogenetic proteins (BMPs), and platelet derived growth factors (PDGFs) have also been demonstrated to be osteogenic stimulators (Behr et al., 2010; Kempen et al., 2010; Jeon et al., 2012). Therefore, scaffolds can be designed to provide biophysical and biochemical cues for stimulating osteogenic differentiation of stem cells and other osteoprogenitor cells. Moreover, this osteogenic differentiation is marked by an up-regulation of transcription factor Runx2, alkaline phosphatase (ALP), osteocalcin (OCN), osteopontin (OPN), bone sialoprotein (BSP), and ECM phosphoglycoproteins, among others, which stimulate mineralisation (Fakhry et al., 2013). Interestingly, it is known that many ions are involved in this process, such as Si^4+^, Sr^2+^, B^3+^, Ca^2+^, and Zn^2+^ and others which will be described more in detail in section Osteogenic Actions (Reffitt et al., 2003; Fukada et al., 2013; Hashimoto, 2020).

### Mineralisation

Mineralisation of the extracellular matrix (ECM) is the last phase of bone healing and consists in a gradual reabsorption of the primary cartilaginous callus and replacement by a hard callus (Dimitriou et al., 2005). The arrival of MSCs to the site of injury activates their differentiation into chondroblasts and the molecular cascade of type I and type II collagen secretion. At this point, the transforming growth factor beta (TGF-β) and BMPs play an important role as they are involved in the chondrogenesis and the initiation of the bone healing cascade (Cho et al., 2002; Tsuji et al., 2006; Marsell and Einhorn, 2009). Consequently, this mineralisation step is the result of multiple interactions between cells, ECM proteins and environmental factors such as pH, ionic strength, and mechanical stimulus (Marsell and Einhorn, 2011).

During mineralisation, osteoblasts secrete matrix vesicles containing small calcium phosphate crystal precursors and lipids that are capable of attracting calcium (Golub, 2009). Moreover, collagen type I (COL1) acts as a template for the precipitation of the inorganic matter (Fedde et al., 1999). Together with COL1, other proteins from the ECM of bone, such as OCN, OPN, BSP, and phosphoglycoproteins, have a key role in the mineralisation process. Some of these proteins have calcium binding domains, which in turn bind to phosphate ions, allowing the formation of apatite crystals (Chen et al., 1992).

## Incorporating Ions Within Biomaterials

Osteogenic ions have been incorporated into biomaterials to enhance bone regeneration. Depending on the type and morphology of the biomaterials, different incorporation techniques can be used that result in different ion release characteristics. In this section, a brief description of the incorporation of ions in different materials and the overall morphology of the composite (or the potential bone substitute), which, in fact, will modulate the ion release, will be described.

### Types of Materials and Incorporation Methods

**Figure 2:**
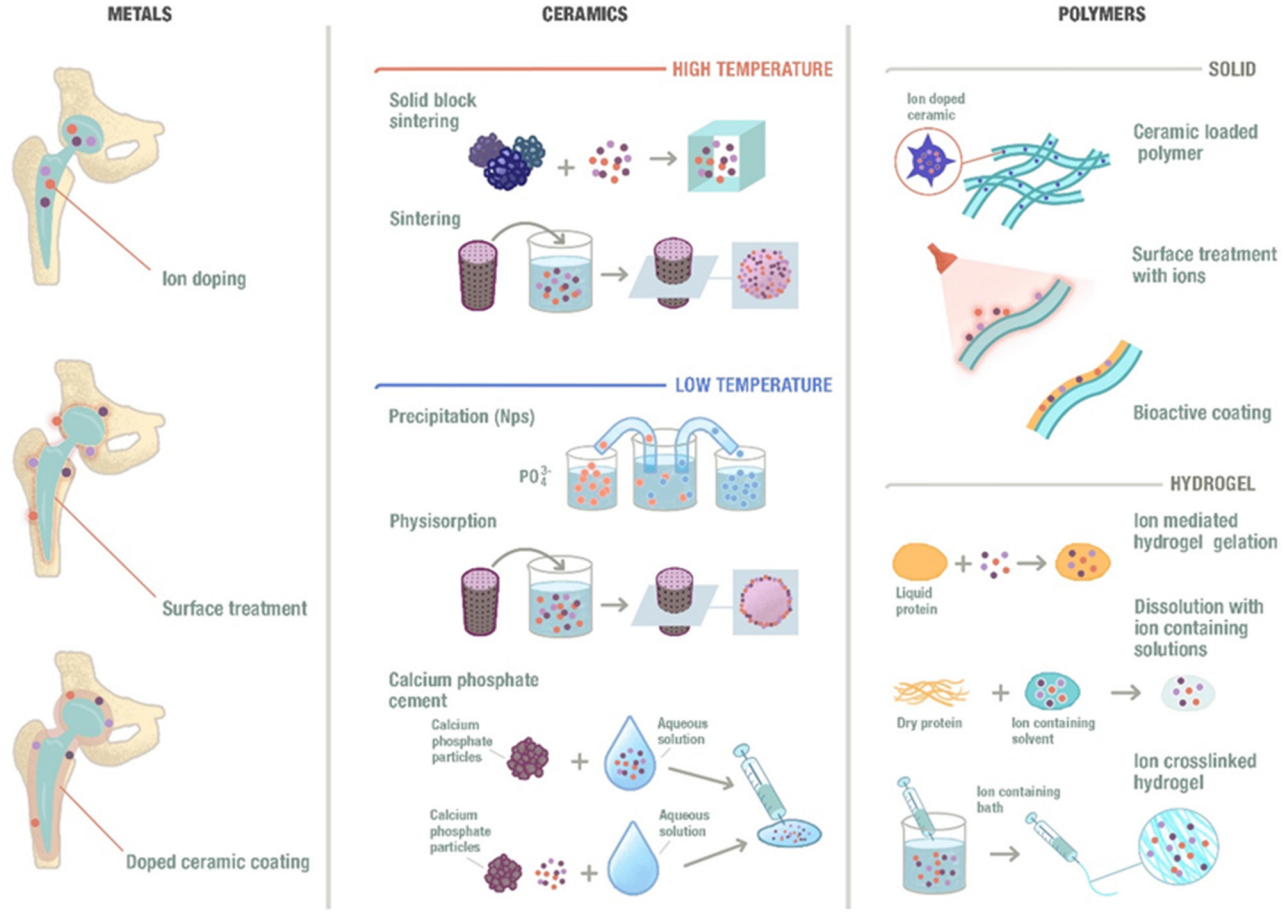
Schematic representation of the ion incorporation strategies depending on the different types of material and morphologies. Ions could be incorporated to metallic biomaterials by integrating them into their composition, performing a substrate treatment or including them into a ceramic coating that will be applied to the surface of the metal. Moreover, ions can also be incorporated into ceramic materials at high temperatures or low temperatures and into polymeric materials following different methods.

An overview of the possible designs of biomaterials incorporating ions is shown in Figure 2, where different types of morphologies are established as well as different types of compositions. The different possibilities will be briefly described.

#### Ceramics

Ceramics are commonly used materials in the field of bone tissue regeneration. A well-known ceramic is hydroxyapatite (HA), which presents multiple synthesis routes at low or high temperature (Habraken et al., 2016). One of the main disadvantages of HA is the low degradation and resorption rates, mainly related with its high degree of crystallinity. In order to reduce its crystallinity, and hence to trigger reabsorption, its structure is usually disrupted by incorporating different ions, such as Sr^2+^, Zn^2+^, or Mg^2+^, among others (Reger et al., 2019). For the high temperature ceramics, these ions are generally incorporated by doping the apatites prior to synthesis. Another strategy to incorporate ions is to sinter the apatites in the absence of the ions, which can then be soaked in different solutions containing the desired ions to allow their physisorption (Garley et al., 2019). For the low temperature ceramics, these ions can be introduced by simply incorporating the desired ion into the calcium and phosphate solutions, forcing its reaction and, consequently, leading to the precipitation of the doped apatite (Evis and Webster, 2011; Ofudje et al., 2019). A slightly different approach for the low temperature fabrication is the use of calcium phosphate cements (CPC), which is based on the mixing of a powder and a liquid to form solid apatite at room temperature (Perez et al., 2012, 2015c). In this case, the ion can be incorporated either as a salt within the powder, or as an ionic solution within the liquid phase (Schamel et al., 2017).

Ion-doped glass-ceramics and glasses have also been used in bone tissue engineering due to their bioactivity, resorbability and appropriate mechanical properties. The incorporation of trace elements modifies their biological activity, allowing the release of therapeutic ions from their structure (Hoppe et al., 2011). The release kinetics of these ions can be controlled depending on the incorporation method used as well as on the physicochemical properties of the material (Castaño et al., 2017).

#### Polymers

The ability of polymeric materials to incorporate ions differs depending on the type of polymer used. Synthetic polymers are less susceptible to easily incorporate metallic ions, since tedious and complicated chemical routes are needed. Other option is the blending of such polymers with ionic-releasing particles, such as bioactive glass (Olmos Buitrago et al., 2015; Alizadeh-Osgouei et al., 2019). Among the different natural polymers that can be used, polysaccharides (such as alginate) and natural proteins (such as collagen) are the most commonly used. Amongst the polysaccharides, alginate is an interesting example of a versatile polymer that allows the incorporation of ions by simple crosslinking of the polysaccharide chains with divalent ions (Perez and Kim, 2013; Perez et al., 2015a). This allows the gelation of the polymer, which maintains the ion within the structure. Once the hydrogel is placed within a monovalent ion solution, the ions are exchanged, releasing the divalent ions within the alginate.

#### Metals

Different ions have also been successfully incorporated to metallic biomaterials. Since ions are generally of metallic origin, these can be integrated within the overall composition of the metals, allowing a slow and sustained release (Alrabeah et al., 2017). While this may take long time to allow the release of the therapeutic ions, the continuous exchange of ions and the liquid environment may lead to certain corrosion issues that need to be controlled (Noumbissi et al., 2019). Furthermore, the ions can be incorporated on the surface of the metallic substrates by surface treatments, such as etching, plasma treatment or coatings (Lu et al., 2012). For example, a ceramic coating can be applied to the surface of the metallic device to incorporate the therapeutic ions (Kose et al., 2013). These ions will mainly be found on the surface and their release may be more easily tuned without jeopardizing the bulk properties of the implant.

### Overall Morphology

**Figure 3:**
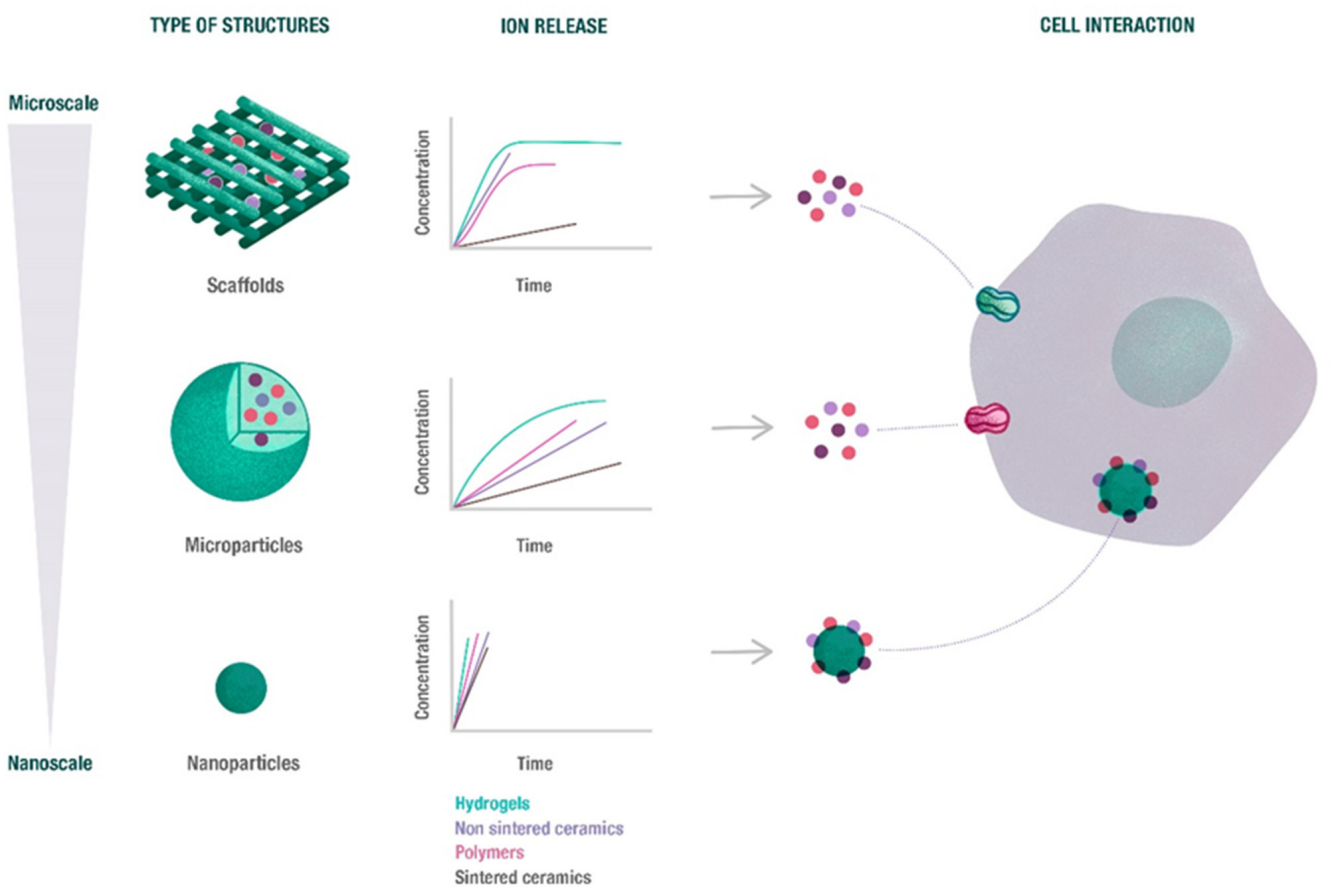
Overview of the potential release of ions depending on the biomaterial morphology. The release profile of ions from scaffolds, microparticles and nanoparticles depends on biomaterial size and morphology, as well as its composition. Nanoparticles rapidly released the ions, while microparticles exhibited a sustained release. Moreover, the ions released from scaffolds and microparticles are able to enter cell through ion channels, while nanoparticles can be directly internalized.

Besides the biomaterial composition, different morphologies of the final device might determine how ions are released (concentration and time) and sensed by the cells, (Figure 3).

#### Scaffolds (Porous and Fibrous)

Three dimensional scaffolds are the most commonly used structures for bone tissue regeneration. There are several requirements for an ideal scaffold for bone tissue engineering, mainly to provide similar mechanical properties to those of the native bone (especially in load-bearing areas), to degrade while the new bone is being formed, to present sufficient porosity allowing cell infiltration, as well as to give osteoconductive and osteoinductive cues for bone maturation (Perez and Mestres, 2016). These scaffolds can be fabricated by several methods, including freeze-drying, solvent casting, solid freeform fabrication or phase separation, among others (Chocholata et al., 2019). A special type of preparation is electrospinning, which allows forming thin layers of nanofibrous materials, mimicking the fibrillar ECM structure (Xue et al., 2019). Its main disadvantage is the limited space for cell migration (i.e., small pore size and interconnectivity) and low mechanical stiffness. To overcome these limitations, 3D printing is receiving significant attraction, due to the ability to fabricate custom-made structures with greater control over the porosity (size, morphology, and interconnectivity) (Murphy and Atala, 2014; Kyle et al., 2017; Jammalamadaka and Tappa, 2018). These scaffolds, however, generally require an invasive surgery since the devices need to be implanted into the site of the defect.

#### Hydrogels

Hydrogels are a special kind of polymer scaffold. They entrap high amounts of water within the polymeric network. Many hydrogels are fully injectable into the site of defect (Drury and Mooney, 2003). This property offers a great advantage since it reduces the risk of infection in surgery, due to their less invasive characteristics. Furthermore, their low temperature processing (mainly at room or body temperature) allows the delivery of biologically active molecules as well as cells (Bai et al., 2018). The most commonly used hydrogels are based on polysaccharides and natural proteins. Their degradation rates are generally fast but can be strategically cross-linked to tune their degradation rates to match with the formation of new bone. Their main disadvantage is the poor mechanical properties which make them more suitable for non-load bearing applications.

#### Microparticles (MPs)

Based on the gaps provided by the packing of spheres, this allows to use microspheres or microcarriers as a matrix that provides sufficient porosity for cell and bone ingrowth (Park et al., 2013; Salerno et al., 2013). Their main advantage is that each microsphere, in the range between 100 and 400 μm can be used to individually grow cells and allow placing an interconnected macro-scaffold with individually seeded microscaffolds into the site of defect. Overall, this would allow for a uniform distribution of cells throughout the site of defect. Furthermore, their homogenous size allows a constant release of the incorporated molecules or ions (Perez et al., 2014; Timin et al., 2018). Moreover, these can be made injectable/printable by simply mixing the microspheres with a proper hydrogel (Levato et al., 2014).

#### Nanoparticles (NPs)

In a similar way to MPs, NPs present unique properties based on their nano sizes. Their range of sizes down to the nano-level confers them high specific surface areas with electrostatic charge that allows their penetration into cells (Vieira et al., 2017). These are generally synthesized by chemical routes and their size ranges between 20 and 200 nm. These particles can be made of polymeric, ceramic or metallic materials. It is worth highlighting that NPs will offer a significant differential effect compared to MPs based on the fact that a number of NPs are able to enter into the cells and release ions within the cell cytoplasm, whereas MPs are only able to release ions outside the cytoplasm (Palombella et al., 2017) (Figure 3).

## Osteogenic Actions

**Figure 4:**
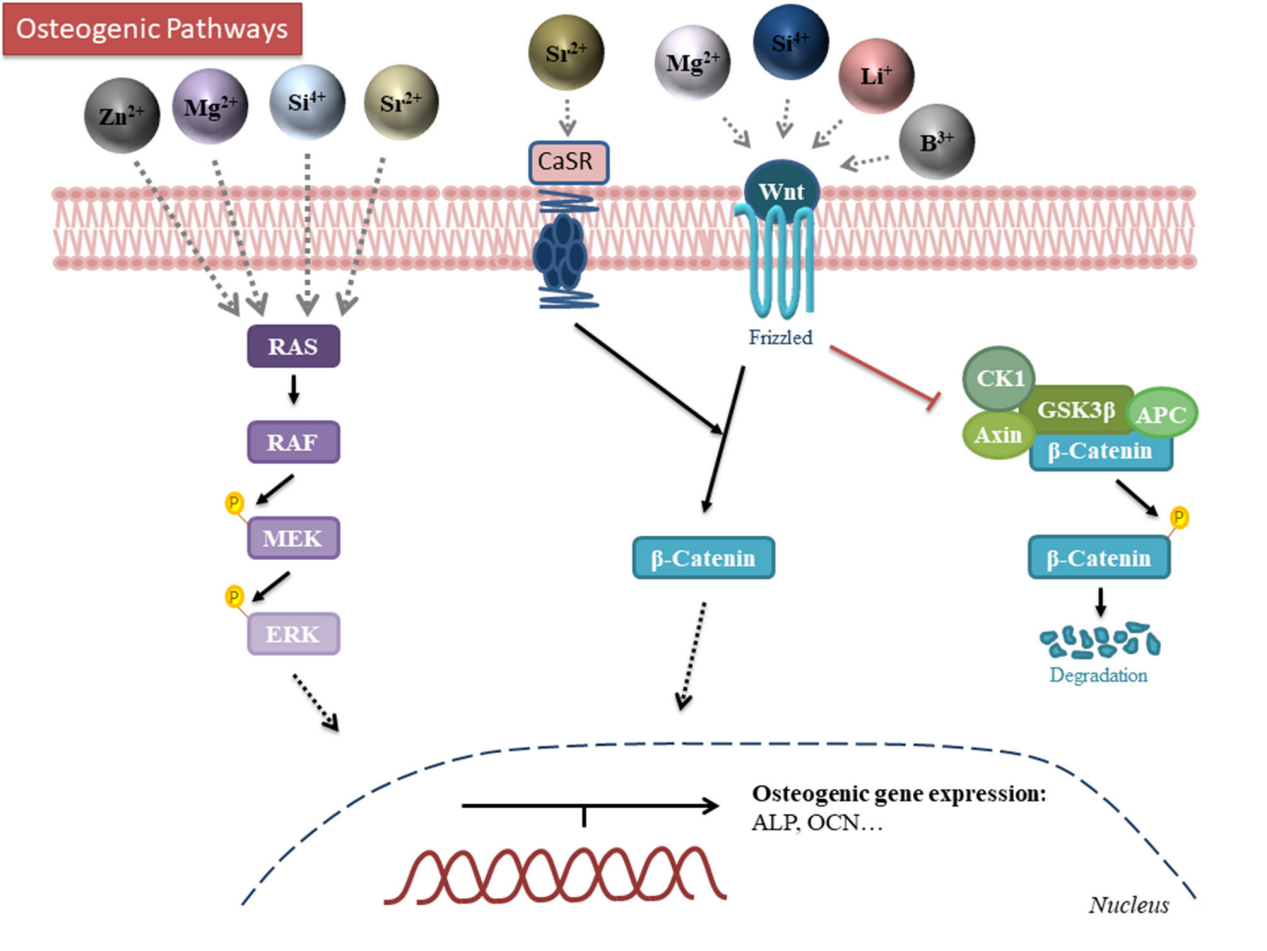
Schematic representation of the mechanism of action for the different osteogenic ions. Zinc, magnesium, silicon and strontium modulate the MAPK-ERK pathway. Strontium, magnesium, silicon, lithium and boron activate the canonical Wnt/β-catenin pathway. Darker color intensity on the ions indicates a more established pathway. B, boron; Li, Lithium; Mg, magnesium; Si, silicon; Sr, Strontium; Zn, Zinc; RAS, Rat sarcoma; RAF, Rapidly activated Fibrosarcoma; MEK, mitogen-activated protein/extracellular signal-regulated kinase kinase (MAPK/Erk kinase); CK1, casein kinase 1; APC, Adenomatous Polyposis Coli.

Some ions are trace elements of bone, being some of them essential for the normal development of the skeleton. Besides, their deficiency can cause diseases such as osteoporosis, in which there is a reduction of the bone mineral mass and change in the microarchitecture (Aaseth et al., 2012). The addition of ions in biomaterials, such as Ca^2+^, Cu^2+^, Sr^2+^, Mg^2+^, Zn^2+^, and B^3+^ amongst others, have shown to enhance osteogenesis and, lead to better overall bone regeneration (Glenske et al., 2018; Lu et al., 2019). For this reason, it is critical to understand how ions interact with cells *in vitro* and how these ions could be incorporated in the appropriate doses within biomaterials to be beneficial both *in vitro* as well as *in vivo*. The main mechanisms of actions of the key ions are schematically represented in Figure 4 and their incorporation into and delivery from biomaterials described below. In order to summarize the different actions promoted by ions, three different summarizing tables are presented. Table 1 shows the effects of the different ions incorporated and released from the structure of metals. Table 2 shows the effects of different ions incorporated and released from ceramics while Table 3 shows the effects of incorporated ions within polymeric materials.

**Table 1:** Summary of studies incorporating different ions within metal biomaterials.

**Type of ion incorporated**	**Type of biomaterial**	**Ion incorporation methodology**	**Content of ion incorporated**	**Ion release rate or amount of ion released**	**Effect on osteogenesis**	**References**
Copper	317L-Cu stainless steel	Ion doping	4.5 wt% of total 317L	0.067 μM per hour	↑ ALP activity ↑ COL1, OPN, Runx-2 ↑ Proliferation ↑ Bone formation	Ren et al., 2015
	317L-Cu stainless steel	Ion doping	4.5 wt% of total 317L	0.137ng/cm^2^/day	↑ ALP and LOX activity ↑ Runx-2, COL1 and OPN expression ↑ Fracture healing	Wang L. et al., 2017
Zinc	Pure zinc rods	Integrated within metal composition	100% Zinc matrix	N/A	↑ ALP staining and activity ↑ Mineralization ↑ ALP, COL1, OPN expression	Zhu et al., 2017
	Zinc ion implanted titanium	Bioactive coating	N/A	1.83–3.98 μM^#^	↑ ALP activity ↑ Mineralization	Jin et al., 2014
	Zinc incorporated TiO2 coating	Bioactive coating	Atomic concentration of zinc: 7.38–15.75 %	3.82–15.30 μM^*^	↑ ALP activity ↑ OCN, ALP, Col I, OCN, Runx2 expression ↑ Mineralization	Qiao et al., 2014
Boron	B-containing coatings on Ti substrates	Surface treatment	0.02 M Na_2_B_4_${\text{O}}_{7^{-}}$10H_2_O	3.7 μM^#^	↑ ALP activity	Ying et al., 2018
Lithium	Lithium coating on titanium scaffolds	Surface coating	0.02 M LiCl	0.43–1.44 μM^*^	↑ ALP, OPN, OCN, COL1, Runx2 expression ↑ Bone ingrowth ↑ Mineralization	Liu et al., 2018

# *Total amount released at the end of the experiment*.

* *The minimum and the maximum amount released during the experiment (based on cell culture media change timepoints)*.

*BMSC, bone mesenchymal stem cells (hBMSC, human; rBMSC, rat); ALP, alkaline phosphatase activity; COL1, collagen type I; OPN, osteopontin; Runx-2, Runt-related transcription factor 2; LOX, lysyl oxidase; OCN, osteocalcin*.

*↑increase*.

**Table 2:** Summary of studies incorporating different ions within ceramic biomaterials.

**Type of ion incorporated**	**Type of biomaterial**	**Ion incorporation methodology**	**Content of ion incorporated**	**Ion release rate or amount of ion released**	**Effect on osteogenesis**	**References**
Copper	Mesoporous bioactive glass	Solid block sintering	5% molar ratio (Copper to Calcium and Silicon)	≈2403 μM^#^	↑ALP, OPN, OCN expression	Wu et al., 2013
	Calcium polyphosphate doped with copper	Solid block sintering	0.1 % molar ratio (Copper to Calcium)	N/A	↑ ALP, OCN expression and calcium node ↑ New bone area	Li Y. et al., 2018
	Bioactive silicate (13–93) doped with copper	Solid block sintering	2 wt% [CuO to silicate (13–93)]	≈ 16–222 μM^*^	↓ proliferation and ALP activity ↓ bone formation	Lin et al., 2016
Cobalt	Hydroxyapatite doped with cobalt	Ionic solution within liquid phase	1 %wt (cobalt to calcium)	0.14 μM^#^	↑ Cell proliferation ↑ Runx-2, Osterix expression	Kulanthaivel et al., 2016
	Hydroxyapatite nanoparticles doped with cobalt	Solid block sintering	12 wt% (cobalt to calcium)	N/A	↑ Mineralization rate ↑ Density and bone surface	Ignjatovic et al., 2015
	Nanoparticles of cobalt-substituted hydroxyapatite	Solid block sintering	5 and 12 wt% (cobalt to calcium)	N/A	↓ Viability ↑ Osteogenesis ↑ Bone density	Ignjatović et al., 2013
Silicon	Mesoporous SiO_2_ microparticles	Ionic solution within liquid phase	100% silicon matrix	≈35–141 μM^*^	↑ ALP activity ↑ COL1, OCN, OPG/RANKL expression	Mao et al., 2017
	Ca–Mg–Si containing bioceramics	Precipitation	N/A	1908–9446 μM^*^	↑ ALP activity ↑ Col I, OCN, ALPase, OPN, OCN expression	Zhai et al., 2013
Zinc	Zinc containing tricalcium phosphate	Sintering	45 mmol ZnCl2 per 100 g TCP	≈ 31 μM^#^	↑ ALP activity ↑ Bone formation	Luo et al., 2014
	Zinc doped mesoporous hydroxyapatite microspheres	Sintering	0.05 molar ratio	≈ 86 μM^#^	↑ Runx2, ALP, OCN expression ↑ New bone area	Yu et al., 2017
Strontium	45S5 bioactive glass	Solid block sintering	50 and 100% molar ratio (Strontium to calcium)	N/A	↑ ALP, COL1, OCN expression	Santocildes-Romero et al., 2015
	HA doped with strontium	Precipitation	10% molar ratio (Strontium to Calcium)	110 μM	No osteogenic effect	Chandran et al., 2018
	Bone ceramic + strontium-substituted nanoHA coating	Physisorption + sintering	10% molar ratio (Strontium to Calcium)	N/A	↑ ALP activity ↑ Runx2, OCN expression ↑ New bone formation	Li J. et al., 2018
	β-TCP + strontium-containing phosphate-based glass (SPG)	Solid block sintering	5, 10, 15 mass fraction (SPG to β-TCP)	≈ 3–14 μM^*^	↑ ALP activity ↑ OPN, OCN expression ↓ osteoclastic activity	Tian et al., 2018
	Strontium-substituted calcium phosphate silicate bioactive ceramic	Solid block sintering	5, 10 and 20% molar ratio (SrCO_3_ to Calcium, Phosphate and Silicon)	457 μM (5%)# 1,484 μM (10%)# 2,968 μM (20%)#	↑ ALP activity ↑ Runx2, OCN, OPN, BSP expression ↑ Mineralization ↑ New bone formation	Zeng et al., 2020
Magnesium	Macroporous HA/Ca/Mg scaffold	Sintering	Immersion in 75000 μM Mg^2+^ solution before sintering	2000 μM^#^	↑ ALP, COL1, OCN, OPN expression	Chu et al., 2018
	Calcium phosphate cement combined with magnesium	Ionic solution within liquid phase	1:2 molar ratio (CaH_4_P_2_O_8_ to MgO)	2000–2500 μM^*^	↑ COL1, ALP, OCN expression	Zhang J. et al., 2015
Boron	B-mesoporous bioactive glass	Solid block sintering	10% molar ratio (Boron to Silicon, Calcium and Phosphate)	≈ 765 μM^#^	↑ COL1, Runx2, ALP, OCN expression ↑ Mineralization ↑ New bone formation	Wu et al., 2011; Yin et al., 2018
Lithium	Lithium doped mesoporous silica nanospheres	Sintering	5% wt (lithium to silicon)	≈ 857 μM^#^ (0.5mg/ml nanospheres)	↑ ALP activity ↑OPN, Runx2, ALP, OCN expression	Zhang et al., 2018
	Lithium-doped calcium phosphate cement	Ionic solution within solid phase	0.3 ml/g of 50 and 100 mM LiCl solution	3,571 μM (50 mM LiCl)^#^ 7,143 μM (100 mM LiCl)^#^	↑ ALP activity ↑ Mineralization ↑ COL1, OCN, OPG, Runx2 ↑ New bone formation	Li et al., 2017

# *Total amount released at the end of the experiment*.

* *The minimum and the maximum amount released during the experiment (based on cell culture media change timepoints)*.

*BMSC, bone mesenchymal stem cells (mBMSC, mouse; hBMSC, human; rBMSC, rat); ALP, alkaline phosphatase activity; COL1, collagen type I; OPN, osteopontin; Runx-2, Runt-related transcription factor 2; OCN, osteocalcin; RANKL, Receptor Activator for Nuclear Factor κ B Ligand; HA, hydroxyapatite; TCP, Tricalcium phosphate; BSP, Bone sialoprotein; OPG, osteoprotegerin*.

*↑increase; ↓decrease*.

**Table 3:** Summary of studies incorporating different ions within polymer biomaterials.

**Type of ion incorporated**	**Type of biomaterial**	**Ion incorporation methodology**	**Content of ion incorporated**	**Ion release rate or amount of ion released**	**Effect on osteogenesis**	**References**
Copper	Chitosan doped with copper	Ion crosslinked hydrogel	62.5 μM per scaffold	N/A	↑ Mineralized tissue formation ↑ Hard tissue ingrowth ↑ Bone volume	D'Mello et al., 2015
	Chitosan and alginate doped with copper nanoparticles	Nanoparticle loaded polymer	0.0635 mg copper NP per mL.	≈1-35 μM^*^	↑ ALP activity ↑ COL1, OCN expression ↑calcium deposition ↑ Bone mineral density ↑ Total bone volume	Lu et al., 2018
Silicon	Silica-hybridized collagen hydrogels	Ionic solution within liquid phase	90:10 and 80:20 weight ratio (collagen to TMOS)	≈ 641–1068 μM^*^	↑ Mineralization ↑ Runx2, OPN, BSP expression	Yu et al., 2015
	Silicon nitride coated Polyetheretherketone	Bioactive coating	N/A	≈ 11–93 μM^*^	↑ ALP, OPN, OCN, Runx2 expression	Xu et al., 2019
	Silicon oxynitride coated wafer	Bioactive coating	N/A	≈ 36–142 μM^*^	↑ Runx2, OCN, COL1 expression ↑ Mineralization ↑ Bone volume formation	Ilyas et al., 2016
Strontium	PCL fibers + Sr carbonate nanoparticles	Ceramic loaded polymer	10 and 20 wt% (Strontium NPs to polymer)	≈ 114–685 μM (10 wt%)^*^ ≈ 23–457 μM (20% wt)^*^	↑ Mineral deposition ↑ BMP-2, Osterix, Runx2 expression	Meka et al., 2016
	Strontium-doped borate BAG particles and chitosan	Ceramic loaded polymer	BAG-Sr: 9% molar ratio (Strontium to BAG ion content) 2g of BAG-Sr in 1ml of chitosan	11–342 μM^*^	↑ Runx2, OCN, BMP-2, COL1, BSP expression ↑ Bone formation ↑ Bone implant contact	Zhang Y. et al., 2015
	Collagen-strontium-substituted HA	Ceramic loaded polymer	Sr-HA: 100% substitution (Strontium to Calcium) 0.2g of Sr-HA in 0.8 ml of collagen	N/A	↑ Bone formation ↑ Deposition of ECM in the bone defect region	Yang et al., 2011
Magnesium	PLA-magnesium particle composite	Mg particles within PLA solution	2 and 5 wt% (Magnesium to PLA)	3680–4590 μM^*^	↑ Cell spreading and mineralized nodules	Zhao et al., 2017
	Bisphosphonate-magnesium NPs within hyaluronic acid hydrogel	Dissolution with ion containing solutions	10, 100 and 250 mM	10.000–100.000 μM^*^	↑ Runx2, ALP, COL1, OCN expression ↑ New Bone Volume	Zhang K. et al., 2017
Boron	Boron doped HA coated chitosan scaffold	Ceramic loaded polymer	10 mg H_3_BO_3_ per 100mL	N/A	↑ COL1 and OPN expression	Tunçay et al., 2017
	Boron-doped chitosan nanoparticles within chitosan scaffolds	Nanoparticles loaded polymer	300 μg Boron in 150 μL scaffold	≈ 1.4–7.4 μM^*^	↑ ALP activity ↑ COL1, OCN, OPN expression	Gumusderelioglu et al., 2015

* *The minimum and the maximum amount released during the experiment (based on cell culture media change timepoints)*.

*BMSC, bone mesenchymal stem cells (mBMSC, mouse; hBMSC, human; rBMSC, rat); ALP, alkaline phosphatase activity; COL1, collagen type I; OPN, osteopontin; Runx-2, Runt-related transcription factor 2; LOX, lysyl oxidase; OCN, osteocalcin; PCL, poly(ε-caprolactone); HA, hydroxyapatite; BAG, bioactive glass; ECM, matrix; PLA, polylactic acid; TCP, Tricalcium phosphate; BSP, Bone sialoprotein; NP, nanoparticles; BMP-2, Bone morphogenetic protein 2*.

*↑increase*.

### Copper

Copper has been described to enhance the osteogenic differentiation in *in vitro* studies. The incorporation of copper in cell culture medium reduced the proliferation of MSCs although it increased the capacity of osteogenic differentiation, inducing an early expression of ALP and mineralisation (Pablo Rodrguez et al., 2002). Little is known on the exact mechanism of how copper affects the osteogenic differentiation. Nevertheless, a possible hypothesis is that it takes place through ECM processing, since several enzymes involved in the crosslinking of collagen and elastin, such as lysyl oxidase (LOX), are copper-dependent, and it is well-known that the ECM composition and structure influence stem cell differentiation (Smith et al., 2018). Some works have also reported a negative effect of copper. A study reported that the supplementation of culture media with copper showed a lower expression of osteogenic genes in rat bone marrow stromal cells (rBMSC), suggesting that copper suppressed the Runx2 signaling pathway which in turn suppressed the collagen deposition (Li et al., 2014). Hence, further studies are needed to elucidate a more realistic and accurate mechanism.

Copper has often been incorporated in metallic materials, as well as in other biomaterials, in order to stimulate bone regeneration. Regarding the metallic materials, several prostheses as well as dental implants present copper in their structure. Interestingly, the presence of copper in dental implants showed increased ALP activity and increased COL1, osteoprotegerin and OPN expression, which together enhanced the mineralisation potential of MSCs (Burghardt et al., 2015). The copper containing implants were then placed in an *in vivo* animal study, showing an increase in bone formation and bone-implant integration (Ren et al., 2015). Similar results were shown for nano-copper-bearing stainless steel, which were shown to increase osteogenic cell expression *in vitro* and to enhance bone formation *in vivo* (Wang L. et al., 2017).

Similar to metals, ceramics have also been doped with copper. Several ceramics, such as bioactive glasses and phosphate-based ceramics, have shown enhanced bone regeneration capabilities. An *in vitro* study showed that copper-containing microporous bioactive glass (Cu-MBG) or its ionic products cultured with human bone marrow stromal cells (hBMSCs) induced a higher expression of ALP, OPN, and OCN (Wu et al., 2013). Similar results were observed *in vivo*, showing enhanced bone regeneration (Li Y. et al., 2018). Furthermore, copper is able to enhance the apatite-forming ability when incorporated into bioactive glass NPs (Zheng et al., 2017). In some cases, copper has been shown to negatively impact osteogenic induction. For instance, pre-osteoblastic MC3T3-E1 cells cultured with bioactive silicate (13–93) doped with different concentrations of copper (0–2 wt%) showed no significant differences in proliferation or ALP activity. Moreover, the highest dose (2 wt% CuO) presented a negative effect in osteogenic response, showing lower ALP activity than the pristine samples. These results were also confirmed with an *in vivo* study in a rat calvaria defect model (Lin et al., 2016).

Unlike metals and ceramics, polymeric materials have rarely been combined with copper ions. Nevertheless, a recent study mixed 2% w/v chitosan solution with 0.625 mM copper solution to fabricate freeze-dried scaffolds containing a total of 62.5 μM of copper within chitosan hydrogels. The resulting doped-scaffolds significantly increased bone volume formation in a rat calvaria defect model, although authors suggest a possible angiogenic role of copper which might benefit bone formation (D'Mello et al., 2015). The main limitation of the study was the fast release of the ions, limiting the presence of the copper ions in the site of defect during the osteogenic induction period. In order to overcome the limitations and the fast release from polymeric materials, copper containing NPs rather than ions have been used. For instance, copper NPs combined with alginate and chitosan significantly increased COL1 and OCN gene expression, as well as the genes involved in cell adhesion, together with an increase in the extracellular calcium deposition. The results were then extrapolated into *in vivo* studies, showing increased bone mineral density and greater total bone volume compared to control (Lu et al., 2018).

### Cobalt

Similar to copper, cobalt is also known to initially stimulate the angiogenic response, which may also contribute to the osteogenic process. Nevertheless, its mechanism is not clearly elucidated. Actually, cobalt-supplemented culture media has not shown a positive osteogenic induction but rather an impairment in the osteogenic differentiation (Patntirapong et al., 2009; Birgani et al., 2016; Drynda et al., 2018). Despite the previous results, cobalt has been incorporated in several biomaterials, mainly in ceramics. For instance, HA powders doped with different concentrations of cobalt (0.5, 1, 5, and 10 w/w%) showed that in samples with 1% cobalt, there was an increase in proliferation of MG-63 cells, as well as an increased expression of Runx2 and Osterix. The proposed mechanism of action was that cobalt acted as a calcium agonist, activating Wingless-related integration site 5 (Wnt5) signaling pathway cascade and hence leading to the expression of Runx2 and subsequently, the expression of Osterix (Kulanthaivel et al., 2016). Positive results were also found *in vivo*, showing that HA NPs doped with cobalt produced dense collagen fibers with a mineralisation rate higher than HA, showing as well a higher bone surface and density in a rat bone mandibular defect (Ignjatovic et al., 2015). However, different results have been found in *in* vitro and *in vivo* studies. For instance, cobalt-doped NPs produced a decrease in viability and cytoskeletal deformation in osteoblastic cells *in vitro*. Interestingly, when implanted *in vivo*, an increased rate of osteogenesis with higher alveolar bone density with a well formed vasculature was found (Ignjatović et al., 2013). The differences between the *in vitro* and *in vivo* results are probably ascribed to the possible negative effects caused at the cellular level by NPs, since it is known that size, shape and morphology may have profound negative effects on cell morphology, which are not necessarily found *in vivo* (Kwon et al., 2013; Perez et al., 2017). With the controversial results found in the literature, it is not clear whether cobalt can be beneficial or useful for osteogenic differentiation. It has been recently hypothesized that the main role of cobalt in bone regeneration is to stimulate angiogenesis with new blood vessel formation, which is an important step to successfully achieve a proper osteogenic differentiation and a proper overall bone regeneration (Perez et al., 2015a; O'Neill et al., 2018).

### Silicon

Another well-known ion that has shown significant effects on osteogenic differentiation and mineralization is silicon. Silicon is found in the active calcification sites in bones and is clearly implicated in the mineralisation process of bone growth (Jugdaohsingh et al., 2002; Han et al., 2013). It was previously suggested that silicon is a key element in the initiation of the pre-osseous tissue mineralisation (Shi et al., 2015). Supplementation with silanol *in vivo* showed that silicon partially stopped the trabecular bone loss in mature ovariectomized rats by minimizing bone resorption and enhancing bone formation, which was hypothesized to be ascribed to the mineralisation of the organic bone matrix stimulation (Hott et al., 1993). The main known mechanism by which silicon interacts with cells is based on the activation of the Mitogen-Activated Protein Kinase-Extracellular signal-regulated kinase (MAPK-ERK) pathway in osteoblast-like cells (Shie et al., 2011). It has also been demonstrated that silicon ions may stimulate cell proliferation and osteogenic differentiation of hBMSCs through the activation of Wnt and sonic hedgehog (SHH)-related gene expression (Han et al., 2013). It has also been demonstrated that silicon promotes *in vitro* osteogenesis in osteoblasts through the activation of mTOR in the PI3K-Akt-mTOR pathway (Zhou et al., 2019). However, another study revealed that the inhibition of mTOR promoted osteogenic differentiation in MC3T3 cells, although this was using strontium ions (Cheng et al., 2019). A possible explanation to this contradiction could be that there are two types of mTOR complexes so they could be playing different roles in osteogenesis (Cheng et al., 2019).

Since silicon is generally present in combination with oxygen, forming silica, the most commonly used strategy for delivering silicon ions is incorporating them in ceramics, generally in the form of scaffolds, either as the bulk materials or as a coating, as well as in the form of NPs. A common strategy to prepare silica based materials is based on the sol-gel reaction of the silica precursors, such as tetraethyl orthosilicate or tetramethyl orthosilicate. Silica based microcarriers are prepared using the silica-based precursors that allowed the hardening of the sol in a water in oil emulsion, producing hardened microspheres after the complete condensation reaction. The microcarriers were shown to allow cell attachment and to induce osteogenic differentiation of MSCs, which was even more enhanced in the presence of other ions, such as calcium (Perez et al., 2014). Similar silica based structures have as well-been incorporated within calcium based ceramics. For instance, calcium-magnesium-silicon-containing bioceramics were compared with β-tricalcium phosphate (β-TCP) ceramics to analyze the osteogenic potential of hBMSCs cultured on the different scaffolds. The proliferation, ALP activity and the protein expression of COL1, ALP, OPN, BSP, and OCN was increased in the silicon based ceramics compared to the β -TCP due to the release of the different ions from the scaffolds. Interestingly, among the three different silicate based scaffolds, which were bredigite Ca_7_MgSi_4_O_16_, akermanite Ca_2_MgSi_2_O_7_ and diopside CaMgSi_2_O_6_, bredigite showed the highest osteogenic potential. The results showed a direct correlation between the scaffolds with the highest osteogenic potential with the amount of silicon ions present (Zhai et al., 2013). Although silicon has been shown to be one of the ions with the highest potential to induce the osteogenic differentiation compared to other ions, its combinations with other ions has been shown to demonstrate even higher synergistic effects. For instance, a bioceramic material containing silicon and strontium components was investigated *in vitro*. The results demonstrated that silicon ions alone had a higher induction effect on ALP activity than strontium ions, and that the combination of both ions resulted on the highest result of osteogenesis. Furthermore, mandibular defects of ovariectomized rat models were tested *in vivo* and demonstrated that bioceramics containing silicon and strontium promoted osteogenesis and mineralisation when compared with β-TCP bioceramics (Mao et al., 2017). This synergistic effect is probably explained by the different activation mechanisms of each ion, since silicon induces the hypoxia inducible factors (HIF) production whereas strontium interacts directly with other membrane receptors (Caverzasio and Thouverey, 2011).

Besides pristine ceramics, composite scaffolds have also been prepared incorporating different silicon-based ceramics within polymer structures. As an example, a siloxane-doped polylactic acid (PLA) and vaterite composite coated with hydroxycarbonate apatite (SPV-H) was used to study the osteogenic activity on MSCs for inducing mineralisation and differentiation. The experimental group was compared with the composite in the absence of silicon using PLA and vaterite as a control (PV-H). In general, MSCs cultured on SPV-H formed bone nodules after 21 days of culture with higher osteogenic differentiation compared to PV-H (Obata and Kasuga, 2009). Another example was observed when silicon ions were incorporated into collagen hydrogels via sol-gel reaction. These collagen/silica hybrid gels were able to increase apatite formation, as well as to induce the expression of Runx2, OPN and BSP osteogenic markers compared to collagen hydrogels (Yu et al., 2015). Moreover, silicon ions could also be incorporated as a coating on several types of surfaces by plasma enhanced chemical deposition. The release of silicon ions from these coatings resulted in an increase of Runx2, OCN, ALP and Col I gene expression *in vitro*. *In vivo* studies further confirmed the osteogenic effect of the coatings, showing the promotion of bone formation and mineralization (Ilyas et al., 2016; Xu et al., 2019).

Apart from the described 3D scaffolds, mesoporous silica NPs have attained great interest due to their ability to encapsulate molecules and hence stimulate different biological processes at the cellular level (Castillo et al., 2019). Silica NPs have been widely synthesized and have shown great potential as delivery cargo carriers for osteogenic induction (Kwon et al., 2013; Perez et al., 2017). These silica NPs are generally in the range of tens of nanometers and have been functionalized by coatings, tethering or co-precipitation of other ions. Readers are referred to previous reviews for a deep understanding of their synthesis and cellular effects (Kwon et al., 2013; Perez et al., 2017).

Silicon-containing glasses have been long used in bone tissue engineering, since their discovery by Larry Hench fifty years ago (Hench, 2006). It has been established that their biological activity comes from the release of biologically-active ions (Baino et al., 2018). Moreover, many new compositions and other types of bioglasses have been proposed for optimizing the body's response according to the specific clinical applications, incorporating other ions such as silver and strontium (Jones et al., 2016).

### Zinc

Zinc is an essential trace metal that promotes osteoblastic proliferation and differentiation, stimulates mineralisation and inhibits osteoclastic cells through promoting bone cell proliferation, ALP activity, and collagen and protein synthesis (Jin et al., 2014; He et al., 2016; Paul et al., 2017). It is known to enhance bone metabolism through 1, 25-dihydroxyvitamin D3, a hormone which regulates calcium action (Yamaguchi et al., 1988; Park et al., 2018). Furthermore, zinc may promote ALP activity and collagen synthesis during osteogenesis. An *in vitro* study analyzed the effect of different zinc containing culture media on osteoblast differentiation in hBMSCs. The results showed that zinc promoted the expression of Runx2 via the cAMP-PKA-CREB pathway, which is an important regulator involved in the osteogenesis of MSCs (Zhu et al., 2017; Park et al., 2018). As previously described, this pathway is known to stimulate the expression of genes such as BMP2, which is well-known to be involved in osteogenesis and bone formation (Kim et al., 2013). Low concentrations of zinc (2 and 5 μg/mL) have also been described to stimulate osteogenesis in rBMSCs through the activation of MAPK-ERK pathway (Yu et al., 2020). However, higher zinc concentrations (15 μg/mL) have been shown to induce cell apoptosis by ROS generation (Yu et al., 2020).

Zinc has been generally added into biomaterials for orthopedic applications as an alternative to other biodegradable metals such as magnesium and iron as well as in ceramic based biomaterials (Zhu et al., 2017; Glenske et al., 2018). Regarding the metallic materials, these have been doped with zinc ions in the field of dental implantology. Titanium implants can be coated with zinc using a plasma immersion technique, which was previously shown to lead to an increased osteogenic activity (Jin et al., 2014). Interestingly, a recent study compared the osteogenic effect of incorporating zinc in titanium with two different coating techniques both *in vitro* and *in vivo*. One method incorporated zinc into the sub-surface of TiO_2_ coatings by plasma immersion ion implantation followed by deposition, whereas the other method consisted of a bulk-doped TiO_2_ by plasma electrolytic oxidation (Qiao et al., 2014). Both the *in vitro* and the *in vivo* results showed enhanced osteogenic behavior on the coating performed by plasma immersion ion implantation. The authors concluded that the samples that incorporated zinc on the surface of TiO_2_ implants, although they had a lower total zinc content, they had the best osteogenic potential and better bone regeneration activity both *in vitro* and *in vivo*, compared to the bulk doped samples. Hence, the results suggest that cells prefer the presence of zinc ions on the surface of the biomaterial rather than zinc ions in the surrounding medium (Qiao et al., 2014).

Nowadays, in a similar trend to other ions, it is becoming more common to incorporate zinc in ceramic based biomaterials. For instance, β-TCP ceramics were doped with three different concentrations of zinc (3, 6, and 9% w/w) with same silicon ion concentration (15% w/w silicon). Biological studies performed with mouse pre-osteoblasts showed adequate cell proliferation and osteogenic differentiation when cells were cultured on the low and intermediate content of zinc (3 and 6% w/w), whereas it was shown that higher zinc content (9% w/w) induced cell death (Paul et al., 2017). The zinc containing ceramics were then implanted into critical sized rabbit femoral condyle defects, showing lower bone formation with the higher zinc concentration (Paul et al., 2017). In a similar way, zinc containing TCP with different concentrations (0, 5, 15, and 45 mmol ZnCl_2_/100 mg TCP) were tested. The results showed that the highest zinc amount promoted the ALP activity of hBMSCs compared to the pristine ceramic. Furthermore, the implantation of the ceramics in the paraspinal muscle of canines for 12 weeks resulted in ectopic bone formation in a dose dependent manner, showing as well no bone formation in the pristine ceramic (Luo et al., 2014). Similarly, the calcium phosphate-based ceramics can be shaped into microsphere with the help of polymeric materials showing as well positive cell osteogenic stimulation in the presence of zinc. Interestingly, the ability to simulate bone regeneration *in vivo* was shown to be highest in the presence of zinc regardless of the presence or absence of collagen and calcium phosphate (Yu et al., 2017).

### Strontium

Strontium is known to increase bone formation and inhibit osteoclast activity and has been proposed as a promising therapy for osteoporosis treatment and prevention (Peng et al., 2009; Aimaiti et al., 2017; Zeng et al., 2020). It has been commonly described that strontium promoted osteogenesis through the activation of the Wnt/β-catenin pathway, increasing Glycogen synthase kinase 3 beta (GSK3β) phosphorylation. This allows two mechanisms: the degradation of phosphorylated β-catenin and the translocation of β-catenin into the nucleus increasing the expression of Runx2 (Yang et al., 2011; Zeng et al., 2020). Another study evaluated that the activation of this pathway was performed through the activation of the calcium-sensing receptor (CaSR), which increases the release of calcium and, subsequently, activates the Wnt signal pathway (Saidak and Marie, 2012). It has also been suggested that strontium activates the Ras/MAPK signaling pathway, subsequently leading to Runx2 transcription downstream (Peng et al., 2009).

Strontium has been mainly incorporated in ceramics or in composite biomaterials. In the case of ceramics, when strontium was added into bioactive glasses, it enhanced the osteogenic gene expression in rat MSCs (Santocildes-Romero et al., 2015). Not only has strontium been able to promote osteogenesis, but also has been shown to reduce the osteoclastic activity (Tian et al., 2018). Their results have not only been shown *in vitro*, but also *in vivo* (Chandran et al., 2018; Li J. et al., 2018). While ceramics are an interesting and an appealing source for bone regeneration, composite materials are as well an interesting type of materials to dope with strontium. A nanocomposite fibrous scaffold made of poly(ε-caprolactone) (PCL) doped with strontium carbonate NPs increased the proliferation of human MSCs, as well as the expression of BMP2, Runx2 and Osterix in the mRNA and protein level, and also increased the deposition of calcium minerals even with the absence of osteogenic factors (Meka et al., 2016). Similarly, strontium doped composites have shown an enhanced bone regeneration (Zhang Y. et al., 2015; Henriques Lourenço et al., 2017). For instance, strontium-doped HA microspheres embedded in strontium-crosslinked RGD-alginate hydrogel were implanted *in vivo* in a rat critical-sized bone defect model and showed increased bone formation, stimulating the cell migration and collagen deposition (Henriques Lourenço et al., 2017).

### Magnesium

Magnesium and its alloys have been used for biomedical applications for over 2 centuries and it has been observed that its degradation products (magnesium particles and ions) enhance new bone formation. Magnesium ions have been shown to increase ALP activity, matrix mineralisation and the expression of differentiation markers such as Runx2, BMP2, OPN, and COL1 in osteoblasts and MSCs (Yoshizawa et al., 2014; Díaz-Tocados et al., 2017; Wang J. et al., 2017; Zhang X. et al., 2017). Magnesium ions have been described to enhance bone formation mainly through the activation of MAPK-ERK pathway and regulate osteogenic gene expression (Wang et al., 2018, 2020; Yan et al., 2019; Qi et al., 2020). Other molecular pathway activated by magnesium ions is Wnt/β-catenin pathway, which phosphorylates GSK3β as described above and results in an increased osteogenic gene expression (Wang et al., 2018; Hung et al., 2019; Park et al., 2019). It has also been determined that magnesium ions are able to stimulate the activation of Notch pathway, which acts as a regulator of osteogenesis although its exact role in this process remains controversial (Engin and Lee, 2010; Long, 2012; Díaz-Tocados et al., 2017). PI3K pathway is also activated by magnesium ions that enter the cell through TRPM7 ion channel, but the exact molecular mechanisms are still unknown (Zhang X. et al., 2017).

When incorporated and released from biomaterials, magnesium ions have been shown to have different effects. For example, magnesium was incorporated into macroporous scaffolds by hydrothermal calcination. The released magnesium ions were able to promote the osteogenic differentiation of MC3T3-E1 pre-osteoblasts. When calcium was incorporated in combination with magnesium, the surface of these scaffolds presented nano-crystal microstructures with topographical and morphological features that could regulate cell adhesion and could increase even more the expression of the osteogenic markers. In the absence of calcium, although these structures were not observed, the surface roughness was increased compared to the unloaded scaffold (Pan et al., 2015; Chu et al., 2018). Magnesium has also been combined with CPC with different ratios of magnesium to calcium. Interestingly, the greater surface roughness and proper wettability of magnesium/CPC combined with a moderate calcium/magnesium ratio allowed the adhesion of fibronectin in a conformation that enhanced cell attachment and promoted the expression of integrin α5β1 in rBMSCs, which is also involved in cell adhesion. Moreover, the presence of magnesium increased the osteogenic differentiation of these cells compared to the pristine based CPC (Zhang J. et al., 2015). Despite its positive results, further experiments are required to determine whether the increased osteogenic differentiation was due to the effect of the released ions or due to the nanotopograhical structure. PLA/Magnesium composites with evenly distributed magnesium particles embedded in the PLA matrix have been developed by mixing pure magnesium powder with particle sizes about 100 μm with previously dissolved PLA. MC3T3-E1 cells were able to adhere to the surface of the composite, showing that the release of magnesium ions from the material promoted matrix mineralisation (Zhao et al., 2017).

Despite the fact that magnesium is generally incorporated in the presence of other ions, its superior activity in terms of cell adhesion and osteogenic differentiation has been widely studied. For instance, magnesium or strontium were incorporated within the structure of layered titanates. This material was developed using hydrothermal alkaline treatment with NaOH to form titanate nanofibers on commercially available titanium discs. The ions were incorporated by ionic exchange with Na^+^ ions. The exchange efficiency of strontium was higher than the possible doses of magnesium, leading to a lower release rate of magnesium ions. Nevertheless, the cell-material interaction results showed that despite its lower concentrations, magnesium promoted to a higher extent the expression of osteogenic markers Runx2, OPN and OCN in MC3T3-E1 pre-osteoblast cell line in a direct cell culture (Song et al., 2018).

Nanoparticle based systems have also shown to be an effective source of magnesium for its sustained release. In this sense, self-assembled bisphosphonate-magnesium NPs were incorporated within a hyaluronic acid based hydrogel. The formation of bisphosphonate-magnesium NPs was done by simple mixing of methacrylated hyaluronic acid, acrylated bisphosphonate and MgCl_2_ at 10, 100, and 250 mM concentrations. Subsequently, the free acrylated groups of the NPs and methacrylated groups in the hyaluronic acid were crosslinked using UV light. In order to verify the osteogenic capacity of the composite hydrogel, MSCs were loaded within the 3D hydrogels, showing that the material promoted *in vitro* mineralisation and an upregulation of osteogenic genes, which was then confirmed *in vivo* by implanting the hydrogel in craniotomy defects created in Sprague Dawley rats (Zhang K. et al., 2017).

### Boron

Boron is a trace element that is essential for several biological processes such as embryogenesis, immune function, cognitive functions and bone growth and maintenance (Nielsen, 2000). Several studies have shown that boron supplementation could have a beneficial effect in bone health, improving bone strength and microstructure (Devirian and Volpe, 2003; Dessordi et al., 2017). Boron ions have been demonstrated to enhance osteogenesis through the activation of Wnt/β-catenin pathway; in particular, osteogenesis stimulated by boron is promoted by a transcription factor (TCF7L2), which is up-regulated by the activation of the Wnt pathway (Yin et al., 2020). Hence, the delivery of this ion from biomaterials could be a promising therapeutic approach for bone regeneration.

For example, boron ions released from boron nitride nanotubes increased protein adsorption, and MSCs attachment, ALP activity and OCN protein content, indicating that the nanotubes were able to induce osteogenesis (Li et al., 2016). Boron containing coatings have also shown promising results regarding their osteogenic potential. One study showed that a boron doped HA coated chitosan scaffold promoted cell adhesion, ECM synthesis and Runx2, OCN and OPN differentiation marker expression in MC3T3-E1 cells, suggesting that they are able to support osteoblastic differentiation and mineralisation (Tunçay et al., 2017). Another study prepared a boron-containing bioceramic coating generated by micro-arc oxidation onto titanium discs and was able to release low concentrations of boron ions that induced an increase in ALP activity (Ying et al., 2018).

Boron has not only been incorporated into coatings, but also has been integrated in scaffolds. For instance, boron containing chitosan NPs blended within a chitosan scaffold were able to increase osteogenic expression of osteoblastic cells cultured on the scaffolds (Gumusderelioglu et al., 2015). Boron has as well-been directly incorporated into bioactive glass scaffolds (Wu et al., 2011). *In vitro* studies using hBMSCs showed that the scaffolds highly activated Wnt/β-catenin pathway, increasing the presence of β-catenin, p-GSK3β and Setd7 at the protein level. To determine the role of Setd7, a knock down of this protein was performed, observing a reduction in the mineralisation activity and in the expression of osteogenic markers ALP and Runx2 when cells were stimulated with B-MBG, suggesting that promoting osteoblast differentiation is a Setd7 dependent process when cells were stimulated with boron (Yin et al., 2018). However, more research is needed to evaluate if other factors are involved during boron-induced osteoblast differentiation.

### Lithium

Lithium is a microelement present in the human body that has been evaluated to enhance osteoblast proliferation and bone formation (Ma et al., 2019). It has been reported that the molecular mechanism stimulated by lithium is through the activation of the Wnt/β-catenin pathway, which promotes osteogenic differentiation enhancing OCN production and increasing ALP activity (Li D. et al., 2018; Huang et al., 2019).

Lithium doped mesoporous silica nanospheres have been created using sol-gel method showing the ability to rapidly release lithium ions within the first 3 days followed by a sustained release until 14 days. Controlled release of lithium increased cell proliferation, differentiation and the expression of Runx2, OCN, OPN, and ALP in rBMSCs compared to bare mesoporous silica nanospheres (Zhang et al., 2018). A lithium containing coating has been used on a scaffold made of entangled titanium wire in order to enhance bone regeneration. The coatings were developed by micro-arc oxidation of the titanium wire using concentrations of lithium chloride of 0.01 M (L1-MAO) or 0.02 M (L2-MAO). The release of lithium ions from L2-MAO coating was higher compared to L1-MAO coating and it was observed that only L2-MAO was able to enhance MG-63 osteoblast adhesion and spreading, ALP activity, and it up-regulated the expression of Runx2, as well as early (COL1A1 and OPN), middle (ALP) and late (OCN) markers of osteogenesis (Liu et al., 2018).

Lithium chloride dissolved in citric acid at different concentrations (0, 50, 100, and 200 mM) has also been combined with a 1:1 mixture of tetracalcium phosphate and dicalcium phosphate to form a CPC (Li/CPC). A lithium release of up to 60 mg/L from Li/CPC increased the proliferation, ALP activity, mineralisation and expression of COL1A1 and Runx2 in MC3T3-E1 cells through the activation of Wnt/β-catenin pathway. *In vivo*, this material increased the formation of new bone compared to CPC and accelerated bone formation in an ovariectomized rat model (Li et al., 2017). Lithium ions have been recently incorporated into bioactive glass NPs, but their effect on osteogenesis still remains unknown and more studies should be performed (El-Kady et al., 2016).

## Conclusions and Future Perspectives

**Figure 5:**
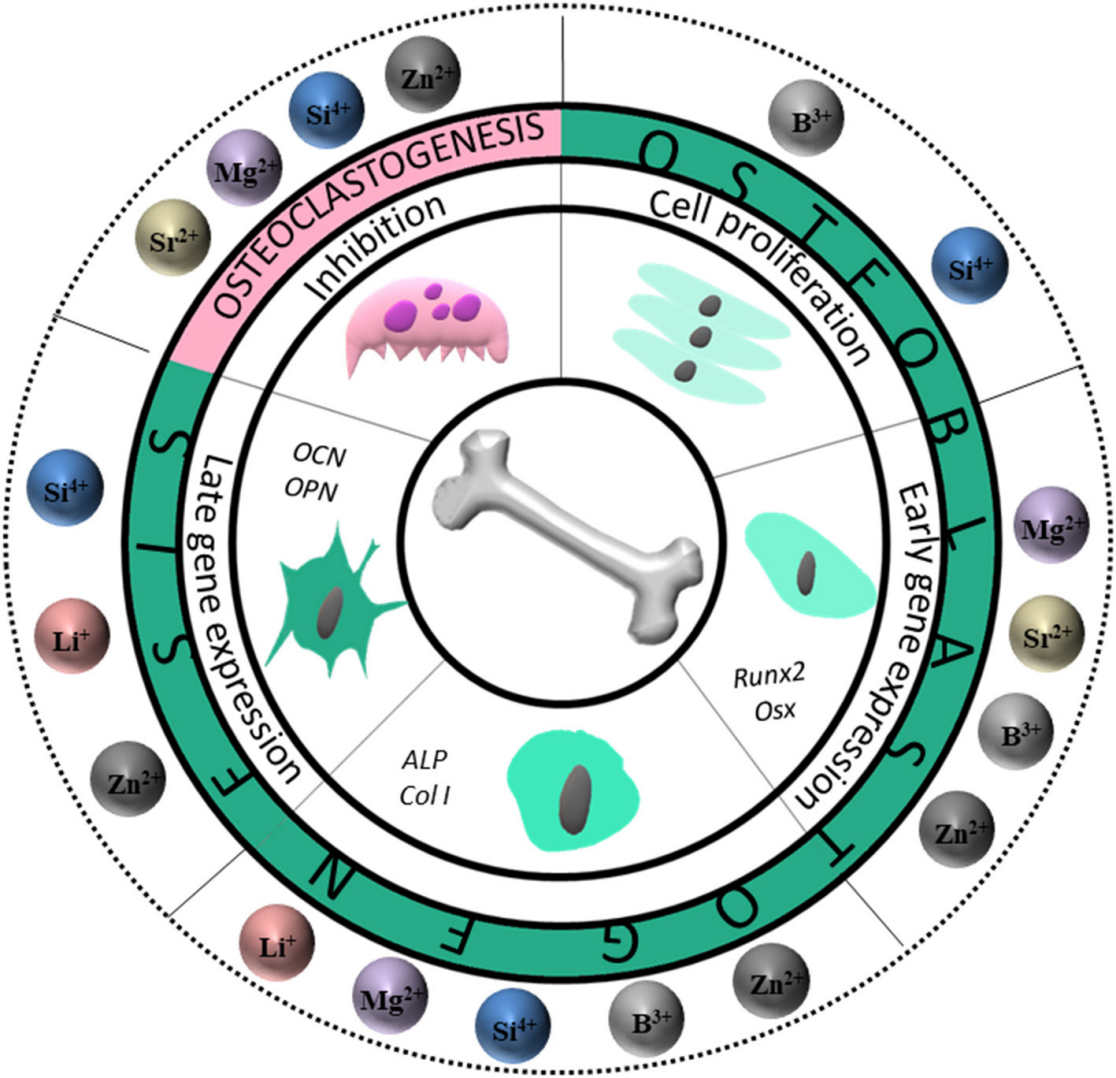
Schematic illustration of the effect of ions during osteoblastogenesis and osteoclastogenesis. First, cell proliferation is stimulated by boron and silicon. Then, zinc, silicon, strontium, boron and magnesium enhance the expression of early genes such as Runx2 and Osx. Later, zinc, boron, magnesium, silicon and lithium stimulate the gene expression of intermediate markers like ALP and COL1. Late gene expression markers OCN and OPN are stimulated by zinc, lithium and silicon. Osteoclastogenesis is inhibited by zinc, silicon, magnesium and strontium. B, boron; Li, Lithium; Mg, magnesium; Si, silicon; Sr, Strontium; Zn, Zinc.

Overall, diverse ions have been shown to have different effects on the different osteogenic actions. Figure 5 summarizes the differential effect for each of the steps considered in bone regeneration, mainly covering the ability of ions to induce cell proliferation, to trigger initial, middle or late osteogenesis, as well as the capacity for the different ions to inhibit osteoclastogenesis.

The recent development of biomaterials that incorporate and release different ions has shown great potential to trigger bone tissue regeneration. While the effective doses vary significantly among these ions, these are still far below the concentrations of growth factors and other molecules needed to produce similar tissue regeneration process. Furthermore, their inorganic nature allows a more stable and prolonged effect compared to other biological molecules. Nevertheless, few similar developments have been established for the sustained and controlled release of ions. For this purpose, further methodologies should be established on how to control on demand ion delivery as well as how to obtain sustained on delivery systems. Currently, significant controversial issues arise from the optimum doses and the release time points, which limits the proper incorporation within biomaterials. Moreover, in order to obtain greater insights of their potential, deeper understating of the signaling pathways involved is needed for the different ions in order to clearly identify the doses required as well as the time points in which these are needed. Overall, the incorporation of ions in biomaterials and their release are promising in providing signaling cues for bone tissue regeneration.

## Author Contributions

EB-R, LD-T, and BG-K contributed to literature search. EB-R, LD-T, BG-K, LD, BB, and MH-N contributed to writing. MM-T, PT, FG, and RP contributed to reviewing and editing the manuscript. All authors have read and agreed to the published version of the manuscript.

## Conflict of Interest

The authors declare that the research was conducted in the absence of any commercial or financial relationships that could be construed as a potential conflict of interest.

## Abbreviations

ALP: Alkaline phosphatase
BMPs: Bone morphogenetic proteins
BSP: Bone sialoprotein
CaSR: Calcium sensing receptor
COL1: Collagen type I
CPC: Calcium Phosphate Cements
ECM: Extracellular matrix
ERK: Extracellular signal-regulated kinase
FGFs: Fibroblast growth factors
GSK3β: Glycogen synthase kinase 3 beta
HA: Hydroxyapatite
hBMSCs: Human bone marrow stromal cells
HIF: Hypoxia inducible factors
MAPK: Mitogen-Activated Protein Kinase
MPs: Microparticles
MSCs: Mesenchymal stromal cells
NPs: Nanoparticles
OCN: Osteocalcin
OPN: Osteopontin
PCL: Poly(ε-caprolactone)
PDGFs: Platelet derived growth factors
PLA: Polylactic acid
rBMSCs: Rat bone marrow stromal cells
SHH: Sonic hedgehog
TCP: Tricalcium phosphate
TGF-β: Transforming growth factor β
Wnt: Wingless-related integration site.
